# Development and validation of automated electronic health record data reuse for a multidisciplinary quality dashboard

**DOI:** 10.1177/20552076231191007

**Published:** 2023-07-28

**Authors:** Tom Ebbers, Robert P Takes, Jimmie Honings, Ludi E Smeele, Rudolf B Kool, Guido B van den Broek

**Affiliations:** 1 Department of Otorhinolaryngology and Head and Neck Surgery, Radboud University Medical Center, Nijmegen, The Netherlands; 2 Department of Head and Neck Oncology and Surgery, Antoni van Leeuwenhoek, Amsterdam, The Netherlands; 3 Radboud Institute for Health Sciences, 213763 IQ Healthcare, Radboud University Medical Centre, Nijmegen, The Netherlands

**Keywords:** Data reuse, structured data, care pathway, quality measurement, electronic health record, dashboard

## Abstract

**Objective:**

To describe the development and validation of automated electronic health record data reuse for a multidisciplinary quality dashboard.

**Materials and methods:**

Comparative study analyzing a manually extracted and an automatically extracted dataset with 262 patients treated for HNC cancer in a tertiary oncology center in the Netherlands in 2020. The primary outcome measures were the percentage of agreement on data elements required for calculating quality indicators and the difference between indicators results calculated using manually collected and indicators that used automatically extracted data.

**Results:**

The results of this study demonstrate high agreement between manual and automatically collected variables, reaching up to 99.0% agreement. However, some variables demonstrate lower levels of agreement, with one variable showing only a 20.0% agreement rate. The indicator results obtained through manual collection and automatic extraction show high agreement in most cases, with discrepancy rates ranging from 0.3% to 3.5%. One indicator is identified as a negative outlier, with a discrepancy rate of nearly 25%.

**Conclusions:**

This study shows that it is possible to use routinely collected structured data to reliably measure the quality of care in real-time, which could render manual data collection for quality measurement obsolete. To achieve reliable data reuse, it is important that relevant data is recorded as structured data during the care process. Furthermore, the results also imply that data validation is conditional to development of a reliable dashboard.

## Introduction

Quality measurement in healthcare is crucial to improve quality of care. However, the process is currently highly time-consuming, because it relies on manual data collection by data extraction employees or healthcare providers. Implementing electronic health records (EHRs) has increased the amount of available data and the opportunities for use of this data for quality measurement. The problem is that most of this data are captured in an unstructured format, which means that the data are not arranged according to a pre-set data model. Therefore, EHR data is currently difficult to reuse for secondary purposes, such as clinical decision support, scientific research and quality measurement.^1^ In most cases, data entry clerks still manually collect data from an EHR to enter into a specific quality measurement database.

To enable automated EHR data reuse for quality measurement, structured data capture is considered critical.^1,2^ Modifying the documentation process is often required to ensure that clinicians document relevant information as structured data. Therefore, the EHR must be adapted to support structured and standardized recording of relevant data at the point of care.^3^ A prerequisite for such a successful transition is that healthcare practitioners are motivated to record data in this structured manner. This can be influenced by various factors, such as perceived ease of use of the EHR and the time required for documentation.^4,5^ Furthermore, several factors can contribute to improving clinician documentation, and therefore structured data recording.^3^ These include sufficiently educating clinicians regarding documentation, standardization of the documentation process to reduce variability, improvement of clinical workflow and ensuring minimal documentation burden by properly aligning structured data capture of the EHR with the clinical workflow.

Once data are recorded in a structured format, the information can be extracted and reused, for example, in quality measurement. However, using automatically extracted data from the EHR is still challenging because of data quality problems.^2,6^ Firstly, these can be caused by problems on the input side of the EHR; this includes a lack of or inconsistent provider documentation, data entry errors (human or computer) or missing coding (such as ICD terminology). Inconsistent provider documentation can have multiple reasons, such as limited skills, insufficient training, time pressure or insufficient user-friendliness of the EHR.^7^ Secondly, the extraction process can cause data quality problems, such as the existence of different places where information is stored (which can also be an input problem), missing coding and incorrect extraction rules. The successful use of EHR-extracted data to calculate reliable quality measures is still challenging, and limited evidence on this topic.^8–10^

In our hospital, a large academic medical center in a metropolitan area, Epic EHR (Epic, Verona Wisconsin) was implemented in 2012. It is the central EHR used by healthcare providers in the hospital. At the Head and Neck Oncology department, with approximately 500 head and neck cancer (HNC) patients per year, the EHR was adapted in 2017 to support routine structured data capture. In the following years, the routinely collected data were used to populate a real-time quality dashboard. This study describes the development of this dashboard in the Head and Neck Oncology department. It also validates the routinely collected structured data used within the dashboard by comparing the EHR-extracted data to manually extracted data. This study aimed to explore whether automated and reliable quality measurement is feasible. We hypothesized that if certain conditions are met, it is possible to use routinely collected data to reliably calculate quality indicators and render manual data collection for quality measurement obsolete. We hoped to gain insight into the quality of the data in the dashboard and whether data quality influences the indicator results. Additionally, this study aimed to learn how data validation can be used to implement data quality improvement cycles.

## Methods

### Automation of data collection and population of the dashboard

**Figure 1.**
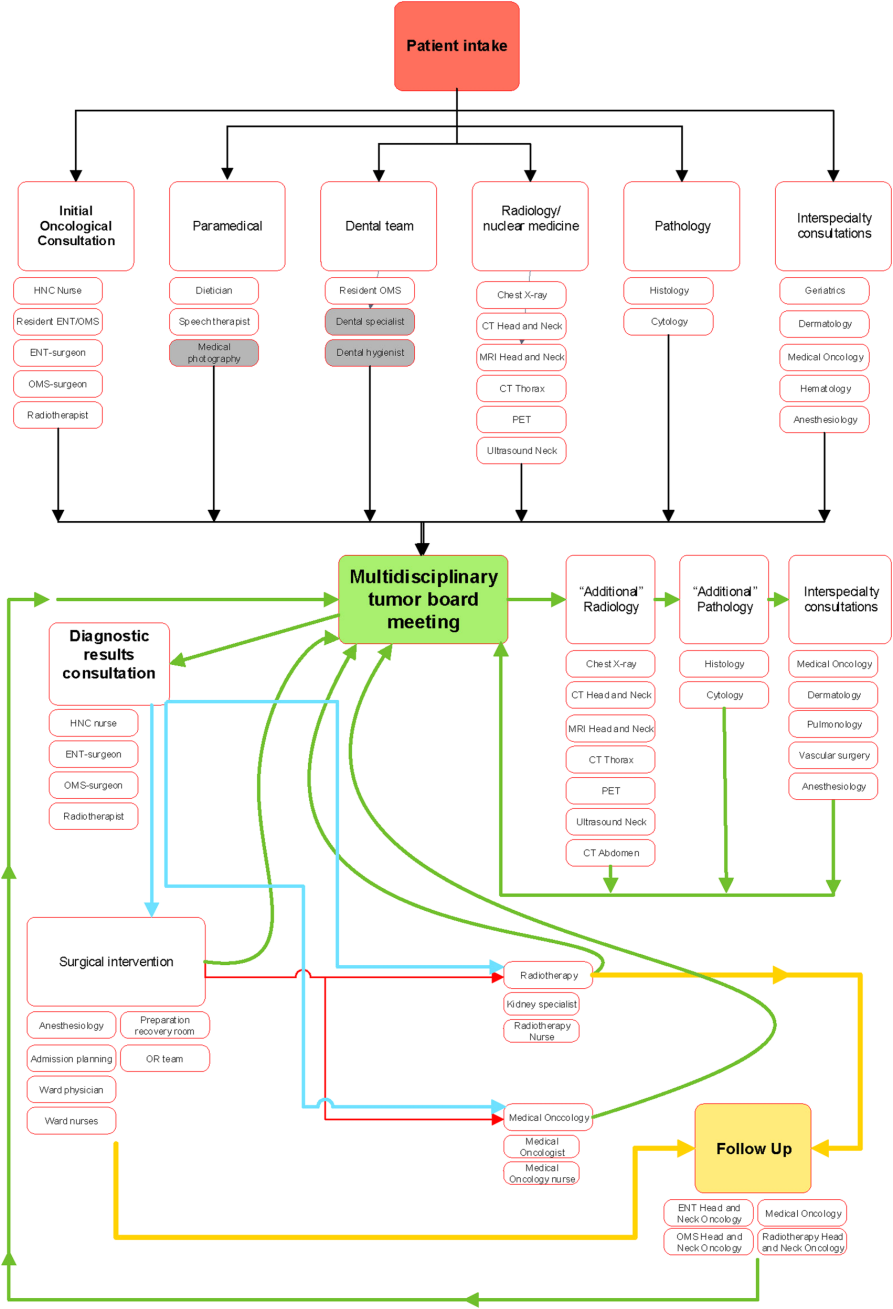
Overview of the head and neck oncology care pathway.

The first step in the development of the dashboard was to determine which clinical variables and indicators should be shown within the dashboard. For this purpose, relevant indicators and clinical variables used in the Dutch Head and Neck Audit (DHNA) were chosen.^11^ The DHNA is the Head and Neck Oncology quality registry in the Netherlands. After reviewing the selected indicators and variables, we defined which data elements were required to calculate these indicators. An indicator consists of a numerator and a denominator and usually has multiple conditions. Therefore, multiple data elements are required for calculating a single indicator. For each data element required, it was considered whether this information was clinically relevant and should be routinely documented by healthcare providers while providing care. If not, this data element should either be captured automatically by the EHR or should not be recorded because this would add to the physician documentation burden. Standardized and structured documentation forms (smartforms) that capture relevant data elements were built and implemented in various phases of care. Care path phases included but were not limited to; triage, diagnostic phase, various treatment phases and follow-up (Figure 1).

These phases usually consist of multiple stops. For example, the diagnostic phase can contain an initial consultation, additional tests and a multidisciplinary tumor board. A smart form was developed for every stop in which relevant data should be recorded. The forms were developed in combination with tools such as automated documentation and standardized, prefilled order sets. As a result, relevant data elements were recorded routinely at the point of care in a structured format and administrative burden was decreased.

This routinely collected structured data were then used to auto-populate the dashboard. The data were extracted daily from the EPIC EHR database using an extraction algorithm. This preliminary data underwent a refinement process using Medimapp Analytics, a third-party business intelligence software (Soulve Innovations, Utrecht, The Netherlands). To illustrate, the software employed specific logic rules to assign specific appointments as the start for various stages in the patient care pathway. For instance, the first radiation treatment appointment code would signal the onset of the therapeutic phase, provided there was no preceding surgical procedure for a specific patient. Additionally, the algorithm was designed to handle situations where multiple multidisciplinary team (MDT) meetings concerning a specific patient were extracted. In these instances, the software, using pre-established logic, identified and selected the correct MDT necessary for the calculation of quality indicators.

Patients were included in the dashboard based on ICD-10 diagnosis codes. For every indicator that was added, extraction logic had to be defined. For example, this included what data elements are required for a specific indicator, their location or locations in the EHR database, and which procedure codes are used in the EHR to indicate relevant appointments, office-based procedures or surgical procedures. Furthermore, the calculation logic for indicators was defined. Because the Head and Neck Oncology care pathway is a complex pathway with many possible variations for a specific patient journey, multiple data validation cycles were conducted by a physician in collaboration with an IT specialist whenever indicators were added to the dashboard. After every cycle, extraction logic and calculation logic were improved. As an example, Appendix A shows the relevant data elements and extraction logic required for one indicator. Appendix B shows a screenshot of the dashboard.

### Validation of automated collected data

To validate the data used in the dashboard, analysis was conducted by comparing the automatically extracted dataset (AED) with the manually extracted dataset (MED) on the same population. The MED was routinely collected by data entry clerks, who routinely collect patient data for the Netherlands Cancer Registry (NCR) and the DHNA.^11,12^ All data used in the dashboard was extracted to create the AED. The inclusion criteria were (a) patients that had an initial consultation for their HNC in 2020; (b) were treated for first primary Head and Neck Squamous Cell (HNSSC) carcinoma (oral cavity, oropharynx, nasopharynx, hypopharynx, larynx, nasal cavity and paranasal sinuses, malignant salivary glands and lymph node metastases of squamous cell carcinoma of unknown origin) and (c) were treated with curative intent. Exclusion criteria were (a) carcinoma in situ; (b) patients diagnosed with a second primary HNSCC carcinoma; (c) a residual or recurrent disease and (d) mucosal melanomas, thyroid tumors, skin tumors, sarcomas, neuroendocrine tumors and hematological malignancies. The MED was requested at the NCR, and the AED was extracted from the dashboard. Inclusion and exclusion criteria were applied to select the study population. Subsequently, records in both datasets were linked based on a unique patient identifier. A linkage indicator was added to the database, which indicated three groups of records: linked records, records uniquely registered in the dashboard and records uniquely registered in the NCR.

### Statistical analysis

Data were notated and analyzed using SPSS version 25 (IBM Corp, Armonk, NY, USA). Percentages were used to assess the level of agreement per variable. Calculations for levels of agreement of the two databases were standardized and therefore applicable on all items, either nominal, categorical or numeric. For date variables, the difference in days between the two datasets was calculated and added as an extra variable. Then, the numerator and denominator of the quality indicators included in this study, the levels of agreement of the results, and the difference in percentage were calculated. Additionally, the kappa statistic was calculated if applicable. Two-tailed significance was defined as *p* < 0.05.

This study did not fall under the scope of the Medical Research Involving Human Subjects Act, which was confirmed by the Institutional Review Board East-Netherlands (2021-13111).

## Results

For both datasets, the total number of patients was counted. The MED contained 330 patients. The unfiltered AED contained 625 patients. After filtering out patients that did not visit the HNC department, 325 patients could be linked, resulting in coverage of 98.48%. After inclusion and exclusion criteria were applied to both datasets, 262 linked records were included for analysis. Table 1 shows the tumor localization of included patients in both datasets, which was based on ICD-10 coding. This variable showed near-perfect agreement with a kappa statistic of 0.96 (*p* = 0.001).

**Table 1.** Tumor localization based on ICD-10 codes for MED and AED.

Localization	Manual *N* (% of total)		Automatic *N* (% of total)	
Oral cavity and lip	82	(31.3)	85	(32.4)
Oropharynx	66	(25.2)	60	(22.9)
Nasopharynx	4	(1.5)	4	(1.5)
Hypopharynx	14	(5.3)	13	(5.0)
Larynx	60	(22.9)	61	(23.3)
Nasal cavity and paranasal sinuses	16	(6.1)	16	(6.1)
Salivary glands	17	(6.5)	18	(6.9)
Unknown primary tumor	3	(1.1)	5	(1.9)
Total	262	(100)	262	(100)

Subsequently, analysis was conducted on the date variables that indicate the start and end of the primary care path phases (triage, diagnostic and various treatment phases) within the Head and Neck Oncology care pathway. The results are shown in Table 2.

**Table 2.** Date variables relevant in mapping the care pathway.

Variable	Records with data present		Records in agreement	
	Manual	Electronic	(If data present in both sets)	Level of agreement
Date of referral	261	257	138/256	53.9%
Date of initial visit	262	258	228/258	88.4%
Date of MDT before treatment	255	259	210/252	83.3%
Date of surgery	132	137	120/122	98.3%
Start date of radiotherapy	199	205	182/197	92.4%
End date of radiotherapy	199	188	172/180	95.6%
Start date of systemic therapy	50	53	39/46	84.7%
End date of systemic therapy	50	53	28/46	60.9%

**Figure 2.**
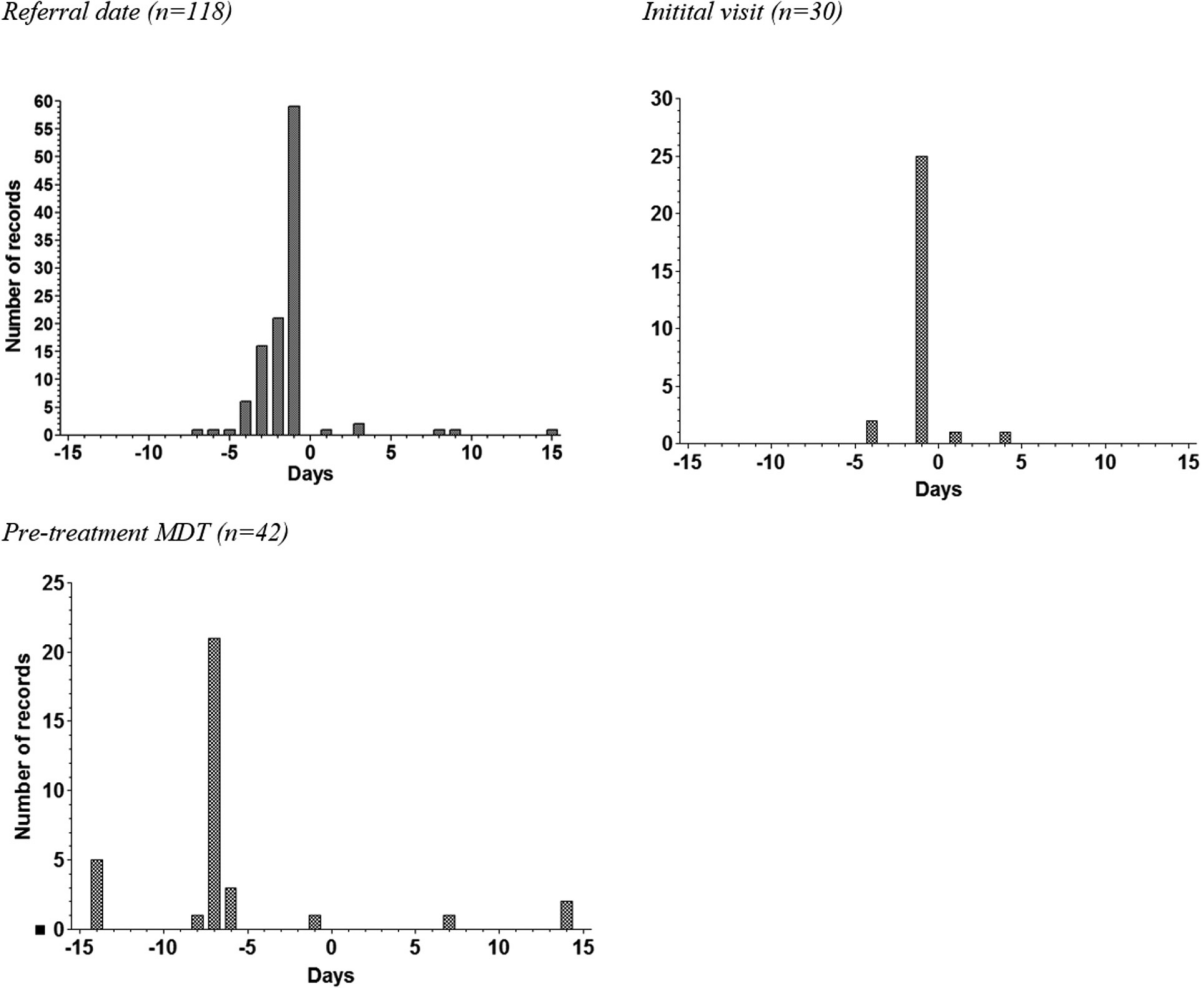
Distribution of calculated difference in manual versus automatically extracted date variables.

The difference in days between the manually extracted date and the automatically extracted date was calculated for records that did not show agreement. Figure 2 shows the distribution of the number of days that the manually extracted date differed from the automatically extracted date for three of these variables. A negative number indicated that the manually extracted date was earlier than the automatically extracted date. A positive number indicates that the automatically extracted date was a date prior to the manually extracted date.

The results of comparing other relevant variables required in calculating the quality indicators, such as treatment modalities, are shown in Table 3.

**Table 3.** Comparison of other required variables for calculating selected quality indicators.

	Records with data present		Records in agreement	
Variable	Manual	Electronic	*(If data present in both sets)*	Level of agreement
Treatment intent	261	262	250/261	95.8%
Tumor localization	262	262	254/262	96.9%
Surgical treatment	262	262	234/262	89.3%
Neck dissection	132	262	115/132	87.1%
Radiotherapy treatment	262	262	248/262	94.7%
Systemic treatment	262	262	251/262	95.8%
Dental team consultation	189	262	183/189	96.8%
Date of dental team consultation	175	179	167/169	98.8%
Physical therapist consultation	43	262	23/43	55.8%
Date of physiotherapy consult	40	22	4/20	20.0%
Surgical complications	132	262	118/132	89.4%
Unplanned reoperation	132	262	128/132	96.7%

After comparing the variables in the datasets, the numerator and denominator of the selected set of indicators included were calculated and compared. The results are shown in Table 4.

**Table 4.** Indicator results (Dutch Head and Neck Audit) - manual versus automatically extracted.

Indicator	Manual clinical quality measurement (*N* = 262) Numerator/Denominator (%)		Electronic clinical quality measurement (*N* = 262) Numerator/Denominator (%)		% difference
Percentage of patients that has been discussed in a MDT prior to curative treatment	242/250	(96.0)	250/261	(95.7)	−0.3
Percentage of patients that started with treatment within 30 days after initial appointment	230/249	(92.4)	232/261	(88.9)	−3.5
Percentage of patients that started adjuvant therapy within 6 weeks after surgical treatment	53/75	(70.6)	59/80	(73.8)	+3.2
Percentage of patients that has had a initial appointment within 7 days after referral	226/248	(91.1)	232/252	(92.1)	+1.0
Percentage of (curative) patients seen by a dental team prior to the start of radiotherapy treatment	167/194	(86.1)	178/204	(87.3)	+1.2
Percentage of (curative) patients seen by a physiotherapist after a neck dissection	40/68	(58.8)	22/63	(34.9)	−24.9
Percentage of (curative) patients that underwent an unplanned re-operation after surgical treatment	9/132	(6.8)	7/136	(5.1)	+1.7
	**Manual clinical quality measurement (*N* = 262) days**		**Electronic clinical quality measurement (*N* = 262) days**		
Median time—initial consultation until MDT	2.0		2.0		=
Median time—referral until MDT	6.0		7.0		+1 days
Median time—referral until initial consultation	5.0		6.0		+1 days

## Discussion

This study described the development and validation of automated reuse of routinely collected structured data within a Head and Neck Oncology care pathway for a real-time quality dashboard. The results show that it is possible to automatically extract highly reliable data from the EHR and use it to calculate quality indicators reliably. The outcomes of these indicators were, in most cases, consistent with results based on manually collected data. This study provides evidence that it is feasible to transition from manual to automatic quality measurement. However, this requires effort to implement structured data recording within a care pathway, enabling electronic extraction and further data reuse. Additionally, continuous data validation is required to improve the quality of the structured data used.

### Interpretation

The coverage of the dataset was nearly 100%. Only five patients were present in the MED while not in the AED. The dashboard used specific ICD-10 codes recorded within Epic EHR to include patients. It was evident that the initial AED contained more patients than the MED. This was because the MED included all patients who met the inclusion criteria of the National Cancer Registry, while the AED was compiled based on the registration date of a subset of ICD-10 diagnosis codes within the EHR. This raw selection of patients requires filtering using additional structured data to get the desired set of patients. Considering the high coverage, this method is feasible, provided that adequate recording of the ICD-10 code is achieved within the care pathway. However, additional filtering should be conducted to obtain reliable denominator of the quality indicators.

The date variables required to map patient care pathways were found to be in substantial agreement, with a rate of up to 98.8%. These variables are primarily used to compute process indicators, specifically waiting times.

Two variables stood out negatively. The date of referral was in agreement in only 53.9% of cases. An explanation might be that during the manual data collection by data entry clerks, the date of referral is found in the referral letter from the general practitioner. This letter is scanned into the EHR as a PDF. Therefore, there is no way to extract the referral date from the EHR automatically. For this reason, during the dashboard development, the administrative assistant's first action, which is the order of scheduling the appointment, was chosen as the referral date. The histogram of the difference between the manually and automatically collected date also shows that there is only 1–3 days difference in most cases. This is likely dependent on the day of referral. As a result of this problem, process indicators which include the referral date in the calculation, showed a median difference of 1 day compared to the MED. Therefore, to increase the agreement on this variable and the indicators using this variable, the EHR should be modified to enable the electronic extraction of the actual referral date, preferably without requiring additional documentation.

For the date of the first consultation, mostly 1-day differences are found. Because there were no apparent explanations for the majority of these 1-day differences, a sample of these patients was checked. The date within the EHR was congruent with the manually extracted date. Subsequently, by consulting with the IT-specialist, an error in the extraction logic was found, resulting in the 1-day differences.

For the pre-operative MDT, it was found that the manually extracted date was often 7 days prior to the automatically extracted pre-operative MDT date. In our hospital, the MDT is conducted weekly and some patients are repeatedly discussed in multiple MDTs. Therefore, it might be assumed that an additional MDT often occurs before treatment starts, which is automatically extracted and marked as the last MDT before treatment. This additional MDT might be missed during manual extraction. Determining which MDT should be used is relevant for specific indicators, such as the median time from first consultation to the pre-operative MDT. Moreover, the definition of existing quality indicators is not always entirely unambiguous and detailed enough, which can result in interpretation differences. Therefore, it is advisable that when transitioning from manual to automatic quality measurement, the definition of existing quality indicators should be revised if necessary, to ensure complete and unambiguous definitions that are computer-interpretable.

Another variable that showed lower agreement was the end date of systemic therapy. Significant differences between the manual and automatically extracted dates were found. In these cases, the extraction logic seems to select systemic treatment end dates of a second round of systemic treatment, probably due to recurrence or residual tumor. This can be attributed to insufficient extraction logic. A solution could be to define a cutoff point in weeks, after which the logic would consider the date part of another round of treatment. For the other relevant variables, a high agreement was found in most variables. However, agreement on whether or not there was a physiotherapy consultation was low. The calculation logic determining whether a physical therapy consultation occurred is based on whether the physical therapist used a specific form. This form is only available for inpatient care, not in the ambulatory setting or when referred to an external physical therapist. This was considered a problem on the input side. On the other hand, when using manual abstraction, data extraction personnel could review a patient record to determine whether they were seen in the outpatient setting, or were referred to an external physical therapist. Consequently, this resulted in low agreement on the indicator using this variable, which was the percentage of patients seen by a physiotherapist after neck dissection. There is a 24.4% difference, with the indicator based on the MED showing 58.8% and the indicator based on the AED only 34.9%. Similarly, multiple studies have shown that automatically extracted EHR data can miss care events.^10,13^

The abovementioned results and examples illustrate that when transitioning to electronic extraction of EHR data and automated quality measurement, the data and the extraction logic should be checked, validated and subsequently improved. Basic validation rules that a manual data extraction employee unconsciously applies are not always incorporated into the extraction logic. Both initial validation sessions during the development phase and subsequent periodic, targeted validation sessions are recommended. Continuing these validation sessions will help improve EHR extraction logic and quality indicator definitions but will also help identify problems or gaps in the structured documentation process. Additionally, clear and unambiguous definitions of quality indicators used are required to ensure reliable and comparable results.

### Comparison with previous research

There is evidence that implementing quality dashboards that provide immediate access to information for clinicians can improve adherence to quality guidelines and may help improve patient outcomes.^14^ However, the quality of data used in these dashboards can be a concern, and calculating reliable quality measures based on EHR data can be challenging.^8,9,15^ Therefore, continuous effort and refinement of all aspects of a dashboard, including data quality, are required to develop a useful dashboard.^6,16^ Studies that compared manually extracted data to automatically extracted data from the EHR found mixed results. One study comparing quality measures based on automatically extracted data from the EHR to manually collected data found low to modest agreement (kappa = 0.36) and an overall disagreement of 30%.^10^ A particular reason was that automatically extracted data frequently missed care events. As a result, some automatically extracted indicators underperformed compared to manually extracted indicators. This is similar to our results regarding the numerator of the indicator physiotherapy consultation after neck dissection, in which the automatically extracted data missed care events and, therefore, the indicator underperformed compared to the manually collected indicator. These findings were supported by a retrospective study stating that workflow and documentation habits can profoundly impact EHR-derived quality measures, and automatically extracted indicators often underperform compared to manually extracted indicators.^13^ Another study investigating the quality of specific automatically extracted variables recorded in preterm births and comparing them to manually extracted variables found relatively high agreement, with discrepancy rates ranging from 3.2% to 12.8%.^17^ A study investigating the relative change of four indicators results based on EHR-extracted data compared to manually extracted data found percentages ranging from 2.4% to 7.2%.^18^ The results of these studies are similar to the results of the variables and indicators compared in this study. Furthermore, these percentages are comparable to discrepancy rates in manual database creation.^19,20^ However, comparison to this study should be made with caution. The quality of data EHR-extracted data and indicators based on EHR-extracted data also varies depending on the characteristics of the data extracted.^21^ For example, structured data such as inpatient medications can be fairly reliably extracted from the EHR, with a median kappa of 0.75 compared to manually abstracted data.^22^ However, when extracting and combining data from different places of the EHR, or data that has been recorded at various points in time is more challenging. Additionally, unstructured data are difficult to reliably extract, and therefore, the quality of automatically extracted data highly depends on the use of structured fields.^10^ Furthermore, data are recorded in the same structured fields over time. When extracting data from a specific field, effort is needed to develop extraction logic that determines the correct value in a specific context. This also influences quality of extracted data. Successful data extraction using natural language algorithms has also been reported, but should mainly be used to enrich structured sources.^23,24^ Overall, a growing body of literature describes that it is feasible to extract structured data from the EHR and use it to calculate reliable quality indicators. Our study confirms these findings and proves it is also possible by extracting routinely collected structured data within multidisciplinary care.

### Strengths and limitations

This study has multiple strengths. We have shown that it is possible to extract structured data in real-time from the EHR and use it for automated quality measures. By comparing this with manually collected data on the same patient group, we can more reliably determine data quality than using other validation methods.^25^ Furthermore, the direct comparison between the manual and automatically extracted data ensured that disagreements could be evaluated. As a result, specific errors in the extraction logic and the structured documentation process can be identified and improved, leading to higher data quality. Lastly, by comparing not only variables but also the results of the quality indicators, it is also possible to gain insight into how significant the impact of disagreement in specific variables is on the result of a quality indicator. Dependent on the indicator, the difference can be considered relevant or irrelevant, which in turn helps to prioritize where the focus of improvement cycles should be or to determine for which indicators data no longer needs to be collected manually.

This study also has some limitations. In this study, we did not conduct a patient-by-patient review of the EHR when a disagreement was found. However, in the development phase of the dashboard, we conducted regular manual validation sessions, cross-checking the automatically extracted data to the EHR. Validation sessions are conditional to data quality improvement in data extraction from EHRs, especially in the initial phase.^26^ In time, targeted, automated validation reports that could show anomalies in the data will be developed and implemented. These will further improve the extraction logic and, if necessary, the documentation process. Another limitation of this study predominantly focused on variables relevant to calculating process and structure indicators, such as date variables for care events. However, when these care events can be reliably extracted for these care event variables, adding clinical variables that are documented during these care events should be possible with minimal extra effort.

### Implications for practice

Current literature describes that measuring quality of care can lead to improved care. Currently, manual data collection for Head and Neck Oncology quality measurement in the Netherlands can take up to 2 hours per patient, and is collected months to even years after initial treatment. Implementing structured and standardized recording and using this data to calculate quality indicators in real-time can speed up the process of quality improvement and reduce costs by making manual data collection obsolete. Furthermore, as more quality indicators and case-mix variables will be added, manual data abstraction of the rising amounts of data will become increasingly time-consuming in the future. However, to use routinely collected data to calculate reliable indicators, the results of our study imply that it is important to standardize the care pathway and the corresponding documentation process.^3^ This reduces variability in documentation practices among different employees, increases the use of standardized vocabulary and reduces the possibility of the same information being recorded in different places in the EHR. These are the main reasons for low data quality when reusing data.^2^ In addition, by periodically reviewing the extracted data and the results in the dashboard, insight can be obtained as to where the gaps in the primary registration process or workflow are, and how the extraction logic and the indicator definitions can be improved. This will increase the data quality within the EHR and the information shown by the quality dashboard. In the future, other functionalities can be developed, such as a signaling function that notifies healthcare providers when a patient is in danger of failing to adhere to process indicators, which is known as a textbook process.^27^ Alternatively, a provider-specific dashboard could be developed that shows the current status of their patients, and where they currently are in their patient journey. Moreover, further steps should be taken to accomplish digital healthcare information exchange with other organizations, such as national quality registries or other healthcare centers. Other clinical information, such as TNM-staging information, should be added to the automatically extracted data from the EHR. Furthermore, To achieve digital information exchange, even more data standardization is needed by using terminology systems and interoperability exchange standards and infrastructures such as HL7 FHIR. Future studies should focus on implementing such infrastructure and validating the data that have been digitally exchanged between institutions.

It is likely that the number of hospitals aiming to implement reusable data capture in the forthcoming years will increase. Currently, the transferability of content to hospitals associated with different EHR vendors presents a challenge. However, our team has contributed to the creation of an implementation manual, a valuable tool for guiding other hospitals in developing and implementing similar care pathways with reusable data capture. It is important to note that healthcare institutions considering similar initiatives should recognize the significant commitment and thorough understanding required to carry out these implementation projects. This includes tasks like mapping out the care pathway, building structured data capture environment within the EHR, defining the extraction logic, data point validation and a multitude of other associated activities. Therefore, a project-based approach is recommended, encompassing healthcare providers, IT specialists and a project leader. Additionally, it would be beneficial to include staff from other relevant areas such as administrative roles and quality assurance personnel. Quantifying the exact human and time resources required for similar projects presents a challenge, given that the process represents a long-term, multifaceted endeavor, engaging various stakeholders over a period of several years. However, the insights gained from this study should provide valuable guidance to other researchers and colleagues who might be undertaking similar initiatives.

## Conclusion

This study provides evidence that it is possible to transition to automatic quality measurement with routinely collected structured data. In most cases, our results showed high levels of agreement between manual and automatically extracted variables and indicator results, but also suggest that continuous validation of data and extraction logic is a prerequisite for reliable results and further improvement of data quality. In addition, the definitions of quality indicators should be unambiguous. The findings of this study should contribute to the development of future EHR-driven quality dashboards.

## Supplemental Material

sj-docx-1-dhj-10.1177_20552076231191007 - Supplemental material for Development and validation of automated electronic health record data reuse for a multidisciplinary quality dashboard Click here for additional data file. Supplemental material, sj-docx-1-dhj-10.1177_20552076231191007 for Development and validation of automated electronic health record data reuse for a multidisciplinary quality dashboard by Tom Ebbers, Robert P Takes, Jimmie Honings, Ludi E Smeele, Rudolf B Kool and Guido B van den Broek in DIGITAL HEALTH

sj-jpg-2-dhj-10.1177_20552076231191007 - Supplemental material for Development and validation of automated electronic health record data reuse for a multidisciplinary quality dashboard Click here for additional data file. Supplemental material, sj-jpg-2-dhj-10.1177_20552076231191007 for Development and validation of automated electronic health record data reuse for a multidisciplinary quality dashboard by Tom Ebbers, Robert P Takes, Jimmie Honings, Ludi E Smeele, Rudolf B Kool and Guido B van den Broek in DIGITAL HEALTH
