# Supplementary material for: Development and validation of automated electronic health record data reuse for a multidisciplinary quality dashboard
Source: Digit Health. 2023 Jul 28;9:20552076231191007. doi: 10.1177/20552076231191007 (PMC10388626; doi:10.1177/20552076231191007)
Supplement: sj-docx-1-dhj-10.1177_20552076231191007 - Supplemental material for Development and validation of automated electronic health record data reuse for a multidisciplinary quality dashboard [file sj-docx-1-dhj-10.1177_20552076231191007.docx]

|  | | |
| --- | --- | --- |
| **Appendix A.** Required data elements and extraction rules required for specific indicator  *Has a patient been discussed in a multidisciplinary tumor board meeting prior to the start of curative treatment?* | | |
| **Information** | **Data elements Extraction logic** | |
| Diagnosis | - Diagnosis code  - Date of diagnosis | ICD-10 code(s) included |
| MDT | - Multidisciplinary tumor board completed Y/N  - Date of MDT | Define appointment code(s) that indicate included MDT(s) Define which MDT should be used if multiple MDT have been conducted |
| Treatment | - Curative or palliative treatment intent | Smart Data Element (location) that indicates curative or palliative treatment intent. |
| Treatment | - Surgical procedure  - Date of surgical procedure | Define procedure codes that indicate (first) surgical treatment Exclude procedure codes for diagnostic procedures Date should be after MDT date |
| Treatment | - Radiotherapy treatment Y/N  - Start date of radiotherapy treatment | Appointment code(s) that indicate (initial) radiotherapy treatment Date should be after MDT date |
| Treatment | - Systemic treatment Y/N  - Start date of systemic treatment | Appointment code(s) that indicate (initial) systemic treatment treatment Date should be after MDT date |
|  |  |  |
