# Supplementary figures and images for: Development and validation of automated electronic health record data reuse for a multidisciplinary quality dashboard
Source: Digit Health. 2023 Jul 28;9:20552076231191007. doi: 10.1177/20552076231191007 (PMC10388626; doi:10.1177/20552076231191007)

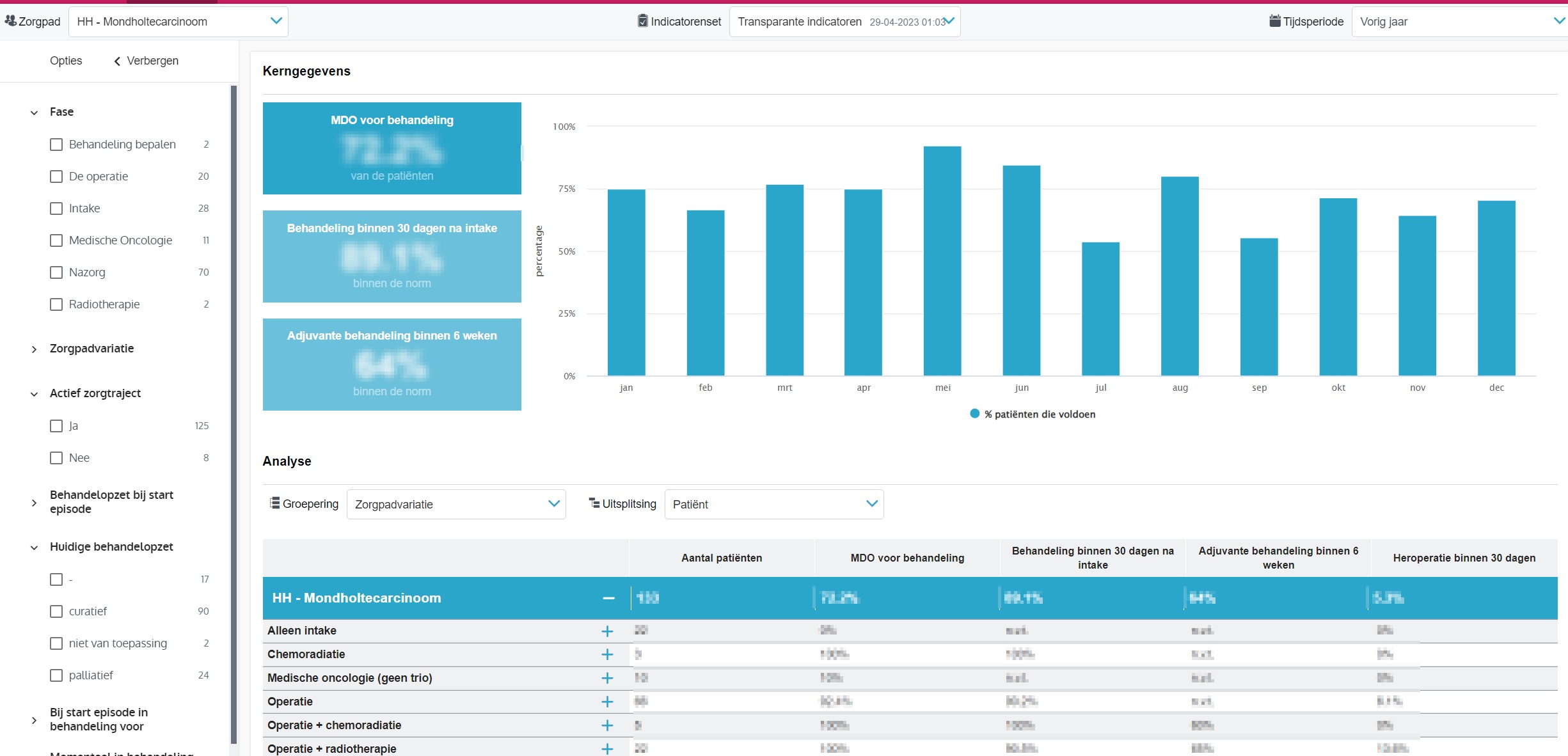

Supplement: sj-jpg-2-dhj-10.1177_20552076231191007 - Supplemental material for Development and validation of automated electronic health record data reuse for a multidisciplinary quality dashboard [file sj-jpg-2-dhj-10.1177_20552076231191007.jpg]
